# Antiviral therapy for hepatitis B virus-related hepatocellular carcinoma after radical hepatectomy

**DOI:** 10.7497/j.issn.2095-3941.2013.03.006

**Published:** 2013-09

**Authors:** Yang Ke, Liang Ma, Xue-Mei You, Sheng-Xin Huang, Yong-Rong Liang, Bang-De Xiang, Le-Qun Li, Jian-Hong Zhong

**Affiliations:** Hepatobiliary Surgery Department, Tumor Hospital of Guangxi Medical University, Nanning 530021, China

**Keywords:** Antiviral therapy, hepatocellular carcinoma, propensity score matching, recurrence-free survival rate

## Abstract

**Objective:**

To assess the effect of antiviral therapy for hepatitis B virus (HBV)-related hepatocellular carcinoma (HCC) after radical hepatectomy.

**Methods:**

A total of 478 HBV-related HCC patients treated by radical hepatectomy were retrospectively collected. Patients in the treatment group (*n*=141) received postoperative lamivudine treatment (100 mg/d), whereas patients in the control group (*n*=337) did not. Recurrence-free survival (RFS) rates, overall survival (OS) rates, treatments for recurrent HCC and cause of death were compared between the two groups. Propensity score matching (PSM) analysis was also conducted to reduce confounding bias between the two groups.

**Results:**

The 1-, 3-, and 5-year RFS rates didn't significantly differ between the two groups (*P*=0.778); however, the 1-, 3-, and 5-year OS rates in the treatment group were significantly higher than those in the control group (*P*=0.002). Similar results were observed in the matched data. Subgroup analysis showed that antiviral treatment conferred a significant survival benefit for Barcelona Clinical Liver Cancer stage A/B patients. Following HCC recurrence, more people in the treatment group were able to choose curative treatments than those in the control group (*P*=0.031). For cause of death, fewer people in the treatment group died of liver failure than those in the control group (*P*=0.041).

**Conclusion:**

Postoperative antiviral therapy increases chances of receiving curative treatments for recurrent HCC and prevents death because of liver failure, thereby significantly prolonging OS, especially in early- or intermedian-stage tumors.

## Introduction

Hepatocellular carcinoma (HCC) is associated with a poor prognosis, and its incidence has increased dramatically in many countries^1^. Hepatectomy is a radical therapy for early-stage HCC. However, even after radical resection, the prognosis for HCC patients remains discouraging because of the high recurrence rate and frequent incidence of intrahepatic metastasis. Therefore, preventing HCC recurrence is very important. Many studies investigated the role of adjuvant and preventive chemotherapies for HCC after radical resection. But until now, there is no definite effective adjuvant or chemotherapies to prevent HCC recurrence^2^^-^^6^.

Hepatitis B virus (HBV) infection is the major risk factor for HCC development in China^7^^,^^8^. Some retrospective studies^9^^,^^10^ have shown that lamivudine treatment for HBV-related HCC patients can effectively reduce the HCC recurrence rate and increase the survival rate after hepatectomy. Other retrospective studies^11^^,^^12^ have shown that postoperative lamivudine treatment neither reduces HCC recurrence rate, nor increases the survival rate of patients, but can reduce the death rate caused by postoperative liver failure. Based on these findings, a meta-analysis^13^ proposes that antiviral therapy based on lamivudine can not only reduce the HCC recurrence rate, but also reduce the death rate caused by liver failure and increase the survival rate of patients. This meta-analysis integrated some retrospective studies with unmatched covariants between groups, otherwise some clinical heterogeneities like different radical therapies, antiviral drugs, dosages, and treatment courses were observed among these studies. Thus, the conclusion from this meta-analysis has limited reliability. Therefore, whether lamivudine antiviral therapy can reduce the postoperative recurrence of HBV-related HCC and extend the survival of patients remains unclear^5^.

To address this issue, we retrospectively collected the clinical data from the prospective database of all HBV-related HCC patients who were treated by lamivudine therapy after radical hepatectomy in our center, and compared with lamivudine-untreated patients in terms of recurrence-free survival (RFS), overall survival (OS), treatments for recurrent HCC, and cause of death. To more objectively assess the effects of antiviral therapy, we conducted propensity score matching (PSM) in the present study.

## Patients and methods

### Clinical data

Inclusion criteria: (1) the initial radical hepatectomy of HCC was performed in the Tumor Hospital of Guangxi Medical University. Diagnosis was verified by postoperative pathology; (2) serum Hepatitis B surface antigen (HBsAg) was positive for all patients; (3) Child-Pugh score was from 5 to 6; (4) Eastern Cooperative Oncology Group score was 0; (5) informed consent was signed.

Exclusion criteria: (1) received transarterial chemoembolization (TACE) or other antitumor therapies before operation; (2) received antiviral therapy (including interferon treatment) in the past year; (3) received prophylactic TACE or other antitumor therapies after operation; (4) combined infection of human immunodeficiency virus, hepatitis C virus, or hepatitis D virus; (5) suffered from other malignant tumors or other severe diseases simultaneously; (6) alcoholism; (7) drug abuse; and (8) pregnant or lactating women.

A total of 478 patients meeting the aforementioned requirements were collected from January 2007 to December 2011. Among these patients, 141 treated by lamivudine after operation were integrated into the treatment group, and the remaining 337 patients without lamivudine treatment were integrated into the control group. To avoid the interference of measured confounding factors, PSM was adopted in making group matches. Exactly 141 pairs were matched successfully. The baseline characteristics of both groups before and after matching are shown in **Table 1**. This research was approved by the Ethics Committee of our hospital.

**Table 1:** Baseline characteristics of treatment and control groups

Covariates	Before propensity matching (*n*=478)				After propensity matching (*n*=282)		
	Treatment group (*n*=141)	Control group (*n*=337)	*P*		Treatment group (*n*=141)	Control group (*n*=141)	*P*
Gender, male/female (%)	129 (91.5)/12 (8.5)	284 (84.3)/53 (15.7)	0.036		129 (91.5)/12 (8.5)	127 (90.1)/14 (9.9)	0.681
Age (years)	48.94±10.47	49.86±11.95	0.432		48.94±10.47	49.70±12.10	0.574
HBeAg, +/- (%)	15 (10.6)/126 (89.4)	41 (12.2)/296 (87.8)	0.636		15 (10.6)/126 (89.4)	16 (11.3)/125 (88.7)	0.849
HBV-DNA (IU/mL)	1.66×10^4^ (0-1.86×10^9^)	3.74×10^3^ (0-3.18×10^6^)	0.041		1.66×10^4^ (0-1.86×10^9^)	1.08×10^4^(0-3.18×10^6^)	0.321
TBIL (μmol/L)	13.51±5.46	14.10±6.93	0.37		13.51±5.46	13.28±6.77	0.753
DBIL (μmol/L)	4.64±2.22	4.86±3.46	0.413		4.64±2.22	4.52±3.59	0.723
TP (g/L)	69.79±6.32	69.70±6.61	0.819		69.79±6.32	70.17±6.63	0.622
ALB (g/L)	40.42±4.57	40.17±4.81	0.599		40.42±4.57	40.58±5.14	0.779
ALT (U/L)	39 (2-504)	35 (2-341)	0.019		39 (2-504)	42 (9-341)	0.878
AST (U/L)	37 (1-270)	38 (1-410)	0.616		37 (1-270)	39 (17-410)	0.96
PT (s)	13.04±1.51	12.86±1.63	0.254		13.04±1.51	13.02±1.78	0.928
AFP, ≥400 ng/mL/<400 ng/mL (%)	38 (27.0)/103 (73.0)	98 (29.2)/238 (70.8)	0.625		38 (27.0)/103 (73.0)	42 (29.8)/99 (70.2)	0.597
Tumor number, 1/2/3 (%)	102 (72.3)/30 (21.3)/9 (6.4)	240 (71.2)/74 (22.0)/23 (6.8)	0.967		102 (72.3)/30 (21.3)/9 (6.4)	107 (75.8)/28 (19.9)/6 (4.3)	0.674
Size of the largest tumor (cm)	4.5 (0.8-15)	5.5 (1.2-26.0)	0.042		4.5 (0.8-15)	5.0 (1.2-14.9)	0.818
Tumor capsule, complete/incomplete (%)	84 (59.6)/57 (40.4)	163 (48.4)/174 (51.6)	0.025		84 (59.6)/57 (40.4)	80 (56.7)/61 (43.3)	0.629
Liver cirrhosis, yes/no (%)	115 (81.6)/26 (18.4)	267 (79.2)/70 (20.8)	0.562		115 (81.6)/26 (18.4)	115 (81.6)/26 (18.4)	1
BCLC stage, A/B/C (%)	107 (75.9)/23 (16.3)/11 (7.8)	256 (76.2)/60 (17.8)/21 (6.2)	0.781		107 (75.9)/23 (16.3)/11 (7.8)	105 (74.5)/26 (18.4)/10 (7.1)	0.882
Follow-up time (months)	24 (2-65)	24 (0-73)	0.057		24 (2-65)	23 (1-73)	0.184

### Methods

Patients of both groups underwent radical hepatectomy for HCC. This procedure followed the Diagnosis management and treatment of HCC (2011 Version)^8^ of the Ministry of Health of the People’s Republic of China. Liver resection technique was performed as previously described^14^^,^^15^. Patients in the treatment group were treated by oral lamivudine after operation. Indications for antiviral therapy followed the Guideline of prevention and treatment for chronic hepatitis B (2010 Version)^16^: (1) for Hepatitis B e Antigen (HBeAg)-positive patients, the value of HBV DNA ≥10^5^ copies/mL (equivalent to 20,000 IU/mL); or for HBeAg-negative patients, HBV DNA ≥10^4^ copies/mL (equivalent to 2,000 IU/mL); and alanine aminotransferase (ALT) ≥ two folds the upper limit of normal; (2) for patients with compensated cirrhosis, HBV DNA ≥10^4^ copies/mL for HBeAg-positive patients, and HBV DNA ≥10^3^ copies/mL for HBeAg-negative patients, which dose not matter whether the level of ALT is high or low; (3) for patients with cirrhosis in the decompensation period, they should receive antiviral therapy once HBV DNA is detected. Lamivudine [Heptodin, from GlaxoSmithKline (China) Investment Co. Ltd.] administration: orally taken by the patients once they had left the hospital or from the first week after the operation for the following one year, with a dosage of 100 mg/d.

### Follow-up and the main outcome measures

Patients were followed up every two or three months after the operation for their serum HBV immune markers, HBV DNA, liver function, prothrombin time (PT), alpha-fetoprotein (AFP), ultrasonography, computed tomography or magnetic resonance imaging, and so on. Postoperative recurrence (including intrahepatic and extrahepatic recurrence) was confirmed through the appearance of intrahepatic or extrahepatic lesions meeting the features of HCC in any imaging examination. The main outcome measures included RFS and OS. Re-treatment measures for tumor recurrence included radical measures like re-operation, radiofrequency ablation, and palliative measures like TACE, systemic chemotherapy, symptomatic and supporting treatment, and so on. For deceased patients, the causes of death including recurrent progress of tumor and liver failure were recorded.

### Statistical analysis

Data analyses were performed using SPSS Statistics 19.0. Variants with normal distribution or approximately normal distribution were described with mean±SD, whereas variants with non-normal distribution were described with median (range). The mean, median, and categorical variant rates between both groups were compared using *t*-test, Mann-Whitney U test, and χ^2^ test, respectively. Survival rate was estimated using the Kaplan-Meier method. Log-rank test was used to compare the differences between the two groups. Differences were considered to be statistically significant if *P*<0.05. PSM was performed using the PSM extension program developed by Felix Thoemmes for SPSS Statistics in 2011^14^.

## Results

### Changes in the balance of baseline material before and after PSM

Five of 18 covariants, namely, gender, HBV DNA, ALT, maximum tumor diameter, and envelope integrity, in baseline characteristics before matching was unbalanced (*P*<0.05). After matching, the covariants were balanced (*P*>0.05, **Table 1**). The balance of baseline characteristics improved based on PSM.

### Cumulative RFS rates before and after PSM

**Figure 1:**
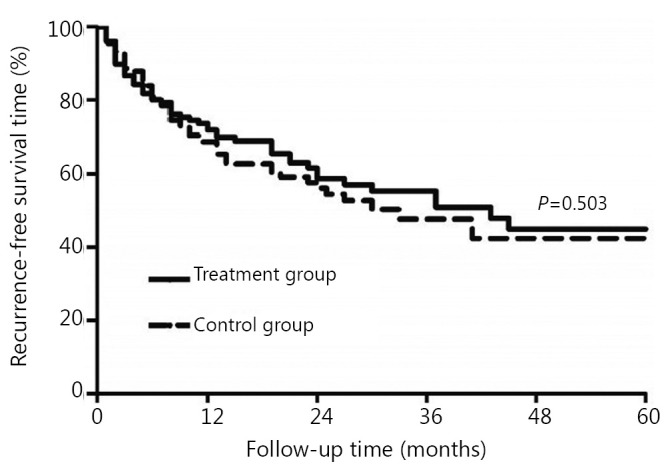
Comparison of cumulative RFS rates between the treatment group and the control group (*P*>0.05) after PSM.

Before matching, the cumulative RFS rates of the treatment (*n*=141) and control groups (*n*=337) at 1-, 3-, and 5-year after the operation were 73.1%, 54.7%, 44.5%, and 70.8%, 58.2%, 52.0%, respectively, *P*=0.778. After matching, the cumulative RFS rates were 73.1%, 54.7%, 44.5%, and 68.8%, 47.8%, 43.0%, respectively (*P*=0.503, **Figure 1**).

### Cumulative OS rates before and after PSM

**Figure 2:**
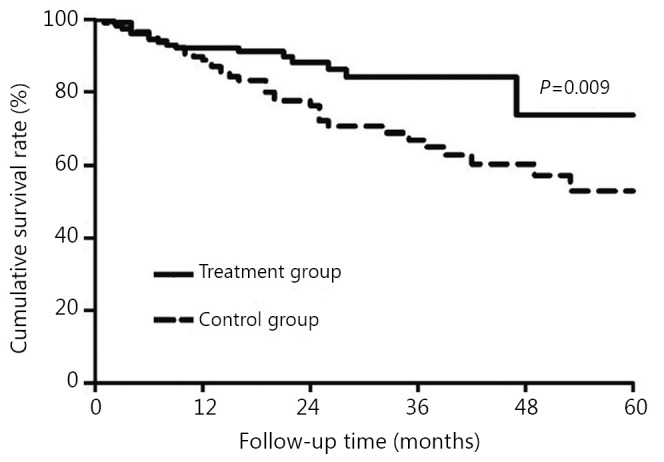
Comparison of cumulative OS rates between the treatment group and the control group (*P*<0.05) after PSM.

Before matching, the cumulative OS rates of the treatment group (*n*=141) and the control group (*n*=337) at 1-, 3-, and 5-year after the operation were 92.1%, 84.4%, 79.1%, and 86.9%, 66.1%, 54.5%, respectively, *P*=0.002. After matching, the cumulative OS rates were 92.1%, 84.4%, and 79.1%, and 89.6%, 66.3%, and 52.1%, respectively (*P*=0.009, **Figure 2**).

### Subgroup analysis of the cumulative OS rates based on Barcelona Clinical Liver Cancer (BCLC) staging

Before matching, the treatment group had 130 cases with BCLC-A/B stages, whereas the control group had 316 cases with the same stages. After matching, the control group had 131 cases, and the cumulative OS rates of patients in the BCLC-A/B stages of the treatment and control groups at 1-, 3-, and 5-year after the operation were 92.2%, 83.9%, 78.5%, and 90.5%, 68.3%, 53.6%, respectively (*P*=0.035). Prior to matching, the treatment group had 11 cases and the control group had 21 cases in the BCLC-C stage. After matching, the control group had 10 cases in the BCLC-C stage, and the cumulative OS rates of patients in the BCLC-C stages of the treatment and control groups at 1-, 3-, and 5-year after the operation were 81.8%, 61.4%, 43.8%, and 80.0%, 48.0%, 48.0%, respectively (*P*=0.775).

### HBV, liver function, tumor characteristics, and re-treatment measures for recurrence

Before matching, 52 and 117 recurrent cases were recorded in the treatment and control groups, respectively. After matching, 57 were noted as recurrence cases in the control group. After PSM, seven indicators, namely, HBV DNA, total bilirubin (TBIL), direct bilirubin (DBIL), ALT, aspartate transferase (AST), PT, and maximum diameter of the recurrent tumor, in the patients of the treatment group were evidently better than those of the control group (*P*<0.05) with recurrent HCC. No differences in intrahepatic recurrence or extrahepatic recurrence, single or multinodular recurrence, and vascular invasion as recurrence between the two groups were observed (*P*>0.05). For recurrent HCC, patients in the treatment group had more opportunities for radical re-treatment (*P*<0.05, **Table 2**).

**Table 2:** Clinical details of recurrent patients in the treatment and control groups

Covariates	Treatment group (*n*=52)	Control group (*n*=57)	*P*
HBV-DNA (IU/mL)	0 (0-4.08×10^6^)	4.36×10^3^ (0-1.46×10^7^)	0.013
TBIL (μmol/L)	12.35 (4.00-113.10)	23.40 (3.40-298.70)	0.04
DBIL (μmol/L)	4.00 (0.40-77.00)	12.20 (2.80-129.80)	0.043
TP (g/L)	70.53±6.51	68.43±6.43	0.093
ALB (g/L)	40.29±4.74	38.81±5.65	0.143
ALT (U/L)	36.50 (9.00-105.00)	42.00 (13-218.00)	0.021
AST (U/L)	35.50 (12.00-121.00)	43.00 (15-344.00)	0.04
PT (s)	13.27±1.38	13.93±1.82	0.032
Recurrence in liver/Recurrence out of liver (%)	48 (92.3)/4 (7.7)	47 (82.5)/10 (17.5)	0.125
Tumor number, single nodule/multiple nodules (%)	24 (46.2)/28 (53.8)	20 (35.1)/37 (64.9)	0.24
Size of the largest tumor (cm)	2.0 (0.4-7.0)	3.2 (1.0-10.3)	0.004
Vascular invasion, yes/no (%)	3 (5.8)/49 (94.2)	7 (12.3)/50 (87.7)	0.399
Retreatment for recurrent HCC, radical treatments/palliative treatments (%)	28 (53.8)/24 (46.2)	19 (33.3)/38 (66.7)	0.031

### Comparison of death causes of patients in both groups

Before matching, 29 and 150 patients died in the treatment and control groups, respectively. After matching, 59 patients died in the control group. Following PSM, 18 (62.1%) and 11 (37.9%) cases died from tumor recurrence and liver failure, respectively, in the treatment group, and 23 (39.0%) and 36 (61.0%), respectively, in the control group (*P*=0.041).

## Discussion

Lamivudine is a type of nucleoside analogue. It can be phosphorylated in cells as lamivudine triphosphate, which is embedded into viral DNA through HBV polymerase by competing with natural substrate deoxyadenosine triphosphate to terminate DNA synthesis. For chronic HBV infection patients, oral lamivudine can inhibit HBV DNA replication, promote ALT normalization, and improve liver inflammation, necrosis, and fibrosis^17^.

Our results showed that lamivudine treatment for HCC after radical hepatectomy could not effectively increase the cumulative RFS rates of patients, but it significantly increased the postoperative OS rates, which was similar to the results of other studies^18^^,^^19^. HCC recurrence includes primary tumor diffusion and multicentric occurrence^7^^,^^20^. Clinically, HCC recurrence within two years after operation is generally regarded as primary tumor diffusion. After 2 years, the recurrence is mostly multicentric occurrence^7^^,^^20^. Randomized controlled trials (RCT)^21^ and a multicentric retrospective study with a large sample size^22^ have shown that lamivudine antiviral therapy can be used for chronic hepatitis B and liver cirrhosis patients to reduce the risk of HCC development. Moreover, HBV infections with continuous high serum titers, as well as continuously or non-continuously increased levels of ALT, are high risk factors of HCC recurrence after radical hepatectomy^23^^,^^24^. So in theory, antiviral therapy can prevent the multicentric recurrence of tumor. However, HCC recurrences in our study mostly occurred within 2 to 3 years after the operation. Such a trend could be attributed to the liver diffusion of the primary tumor and a short follow-up time. This trend could possibly be the important reason for the lack of statistical difference in the cumulative RFS rates between the treatment and control groups, which is the common limitation of this study and other similar studies^11^^,^^12^^,^^18^^,^^19^. To confirm the effect of lamivudine on the recurrence rate, a clinical study with a larger sample size and a longer follow-up period is warranted.

In fact, the first RCT involving 163 patients investigating the efficacy of adjuvant nucleoside analogs to inhibit HBV-related HCC recurrence after radical hepatectomy has been reported^25^. In this trial, all patients had serum HBV-DNA concentrations >500 copies/mL and had Child-Pugh class A or B liver function. In the treatment group, 81 patients were given oral lamivudine (100 mg per day) within 1 week after hepatectomy until HBsAg seroconversion. After a median follow-up of 40 months, RFS was found to be significantly higher in the treatment group than in the control group at both 2 years (55.6% *vs.* 19.5%, *P*<0.001) and 4 years (37.3% *vs.* 12.1%, *P*<0.001). The treatment group also showed significantly higher OS at both 2 years (93.8% *vs.* 62.2%, *P*<0.001) and 4 years (86.4% *vs.* 47.4%, *P*<0.001), as well as a lower rate of early HCC recurrence (44.4% *vs.* 80.5%, *P*<0.001).

Our study introduced BCLC staging into assessment of antiviral efficacy. Patients in the BCLC-A/B stages treated with lamivudine after HBV-related HCC radical hepatectomy obtained obvious benefits in their survival (*P*<0.05). However, patients in the BCLC-C stage treated with oral lamivudine experienced insignificant effects (*P*>0.05). This result could be related to the poorer prognosis of patients in the BCLC-C stage, and failure to reflect antiviral efficacy in the short survival time.

When HCC recurrence was diagnosed, patients in the treatment group had lower HBV DNA level and better liver function indices than those in the control group (*P*<0.05). Most patients had the option of selecting re-operation, radiofrequency ablation, and other radical measures (*P*=0.031). The number of patients who died from liver failure in the treatment group remarkably decreased (*P*=0.041), which was similar to the results of other studies^11^^,^^12^^,^^19^. This result could be attributed to the ability of lamivudine to inhibit or remove HBV to the maximum, ease liver inflammation and fibrosis, impede and halt the progression of liver disease, reduce and prevent hepatic decompensation^17^. With liver function improvement after antiviral therapy, patients could obtain more opportunities for radical treatment and bear a larger resection range at recurrence, and the occurrence of liver failure events decreased, which was translated to the survival of patients.

Our study used PSM to reduce pre-existing differences between the groups, and the balance that is expected to create by a RCT design is here established through statistical matching. The results obtained were more reliable than those from previous retrospective studies^9^^-^^13^. Given the limited conditions, the complete information on the resistance rate of the virus and gene mutation rate were not collected. However, the aforementioned factors might be related to the recurrence of HBV-related HCC and the long-term survival of patients.

In summary, this study showed that the application of antiviral treatment with lamivudine after radical hepatectomy of HBV-related HCC could not effectively increase the RFS rate of patients. However, it provided patients with more opportunities for radical treatment during recurrence and reduced the occurrence of liver failure, which significantly enhanced the postoperative OS rates of patients. Antiviral treatment with lamivudine should be considered after radical hepatectomy for HBV-related HCC, particularly in early and intermedian stage. More RCTs with large sample size and long-term follow-up are warranted to prove our results.
